# Supplementary material for: ACVR1 R206H cooperates with H3.1K27M in promoting diffuse intrinsic pontine glioma pathogenesis
Source: Nat Commun. 2019 Mar 4;10:1023. doi: 10.1038/s41467-019-08823-9 (PMC6399349; doi:10.1038/s41467-019-08823-9)
Supplement: Supplementary file 1 — Supplementary Information [file 41467_2019_8823_MOESM1_ESM.pdf]

Supplementary Information

ACVR1 R206H cooperates with H3.1K27M in promoting Diffuse Intrinsic Pontine Glioma pathogenesis

Hoeman et al.

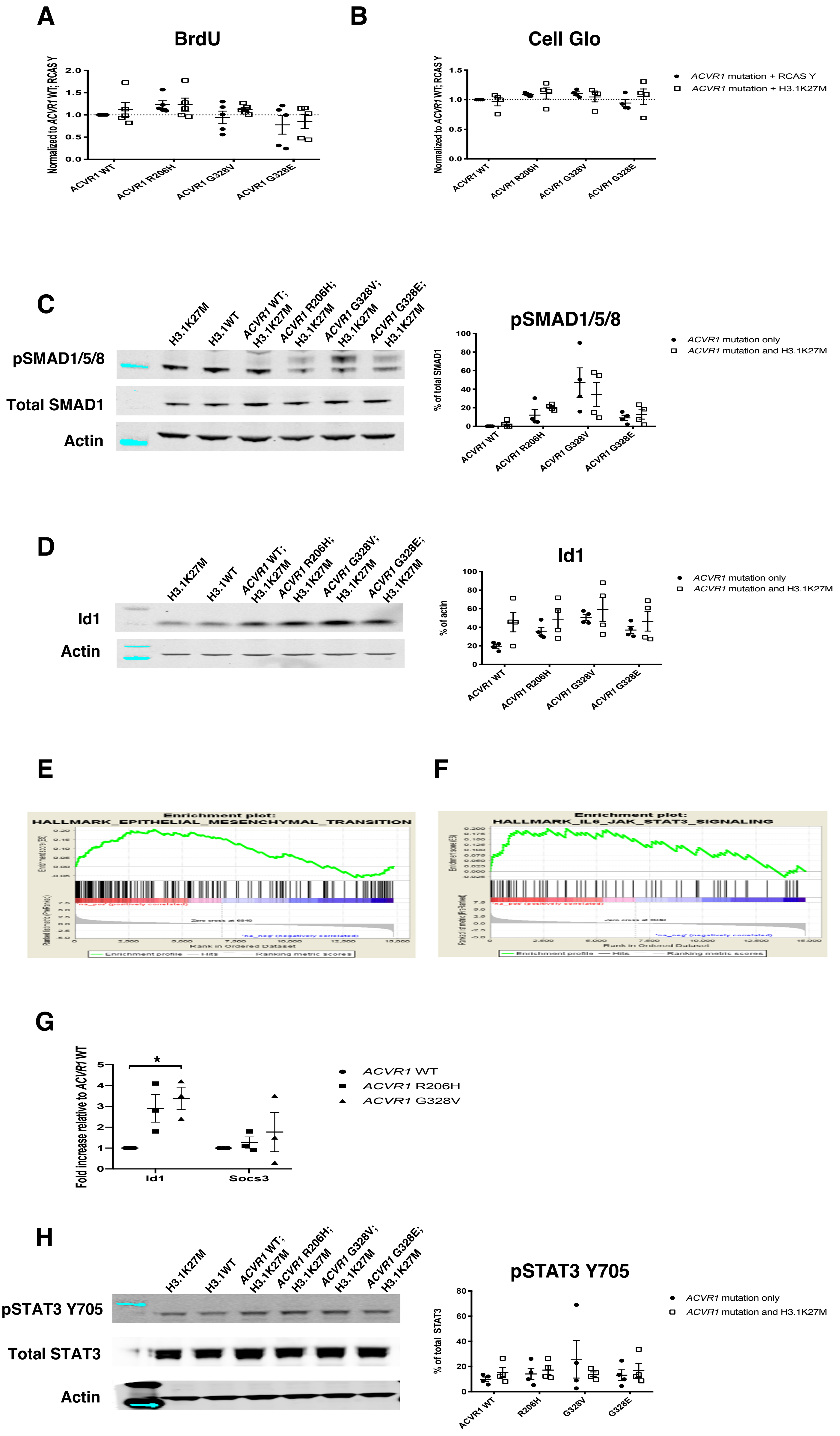
A
BrdU
Normalized to ACVR1 WT; RCAS Y
2.0
1.5
1.0
0.5
0.0
ACVR1 WT
ACVR1 R206H
ACVR1 G328V
ACVR1 G328E
B
Cell Glo
Normalized to ACVR1 WT; RCAS Y
1.5
1.0
0.5
0.0
ACVR1 WT
ACVR1 R206H
ACVR1 G328V
ACVR1 G328E
ACVR1 mutation + RCAS Y
ACVR1 mutation + H3.1K27M
C
H3.1K27M
H3.1WT
ACVR1 WT; H3.1K27M
ACVR1 R206H; H3.1K27M
ACVR1 G328V; H3.1K27M
ACVR1 G328E; H3.1K27M
pSMAD1/5/8
Total SMAD1
Actin
pSMAD1/5/8
% of total SMAD1
100
80
60
40
20
0
ACVR1 WT
ACVR1 R206H
ACVR1 G328V
ACVR1 G328E
ACVR1 mutation only
ACVR1 mutation and H3.1K27M
D
H3.1K27M
H3.1WT
ACVR1 WT; H3.1K27M
ACVR1 R206H; H3.1K27M
ACVR1 G328V; H3.1K27M
ACVR1 G328E; H3.1K27M
Id1
Actin
Id1
% of actin
100
80
60
40
20
0
ACVR1 WT
ACVR1 R206H
ACVR1 G328V
ACVR1 G328E
ACVR1 mutation only
ACVR1 mutation and H3.1K27M
E
Enrichment plot:
HALLMARK_EPITHELIAL_MESENCHYMAL_TRANSITION
F
Enrichment plot:
HALLMARK_IL6_JAK_STAT3_SIGNALING
G
Fold increase relative to ACVR1 WT
5
4
3
2
1
0
*
Id1
Socs3
ACVR1 WT
ACVR1 R206H
ACVR1 G328V
H
H3.1K27M
H3.1WT
ACVR1 WT; H3.1K27M
ACVR1 R206H; H3.1K27M
ACVR1 G328V; H3.1K27M
ACVR1 G328E; H3.1K27M
pSTAT3 Y705
Total STAT3
Actin
pSTAT3 Y705
% of total STAT3
80
60
40
20
0
ACVR1 WT
R206H
G328V
G328E
ACVR1 mutation only
ACVR1 mutation and H3.1K27M

Supplementary Figure 1. ***ACVR1*** **mutations and H3.1K27M do not have an additive effect on proliferation, cell survival, BMP, or STAT3 signaling *in vitro*.** Brainstem progenitor cells isolated from postnatal day 3 Ntv-a;p53$^{fl/fl}$ pups were cultured as neurospheres and infected with RCAS viruses *in vitro* as indicated. (**a** and **b**) Proliferation **(a)** and cell viability **(b)** of neurospheres infected with *ACVR1* and H3.1K27M mutation viruses (white squares) compared to control neurospheres infected with *ACVR1* mutations and RCAS Y (black circles), (n = 4). (**c** and **d**) Western blot analysis of pSMAD1/5/8 (**c**) and Id1 (**d**) of neurospheres infected with *ACVR1* and H3.1K27M mutation viruses (white squares) compared to neurospheres infected with *ACVR1* mutations only (black circles, represented in Fig. 1c and d), (n = 4). (**e**) GSEA analysis of Epithelial to Mesenchymal Transition genes as identified by RNA-Seq analysis (see Supplementary Data 1) in neurospheres infected with *ACVR1* WT or *ACVR1* R206H. Also see Supplementary Data 2. (**f**) GSEA analysis of IL6_JAK_STAT3_signaling pathway as identified by RNA-Seq analysis. Also see Supplementary Data 2. (**g**) qRT-PCR analysis of Id1 and Socs3 in *ACVR1* WT, *ACVR1* R206H, or *ACVR1* G328V infected neurospheres (n = 3), * p = 0.05. (**h**) Western blot analysis of pSTAT3 Y705 of neurospheres infected with *ACVR1* and H3.1K27M mutation viruses (white squares) compared to neurospheres infected with *ACVR1* mutations only (black circles, represented in Fig. 1i), (n = 4). All data are represented as mean with SEM and analyzed using paired t tests.

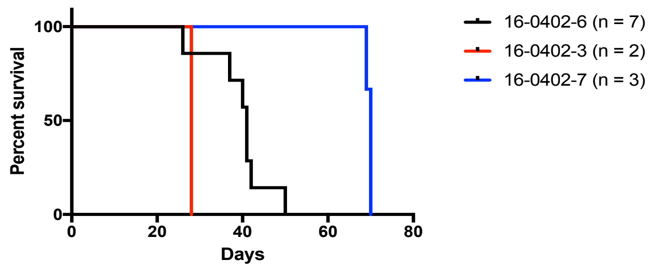

Supplementary Figure 2. *ACVR1* R206H and H3.1K27M are able to generate secondary tumors *in vivo*. Survival curve of Ntv-a;p53$^{fl/fl}$ mice that were implanted with neurospheres derived from *ACVR1* R206H, PDGFA, H3.1K27M, and Cre primary tumors (n = 3 biologically different neurosphere lines).

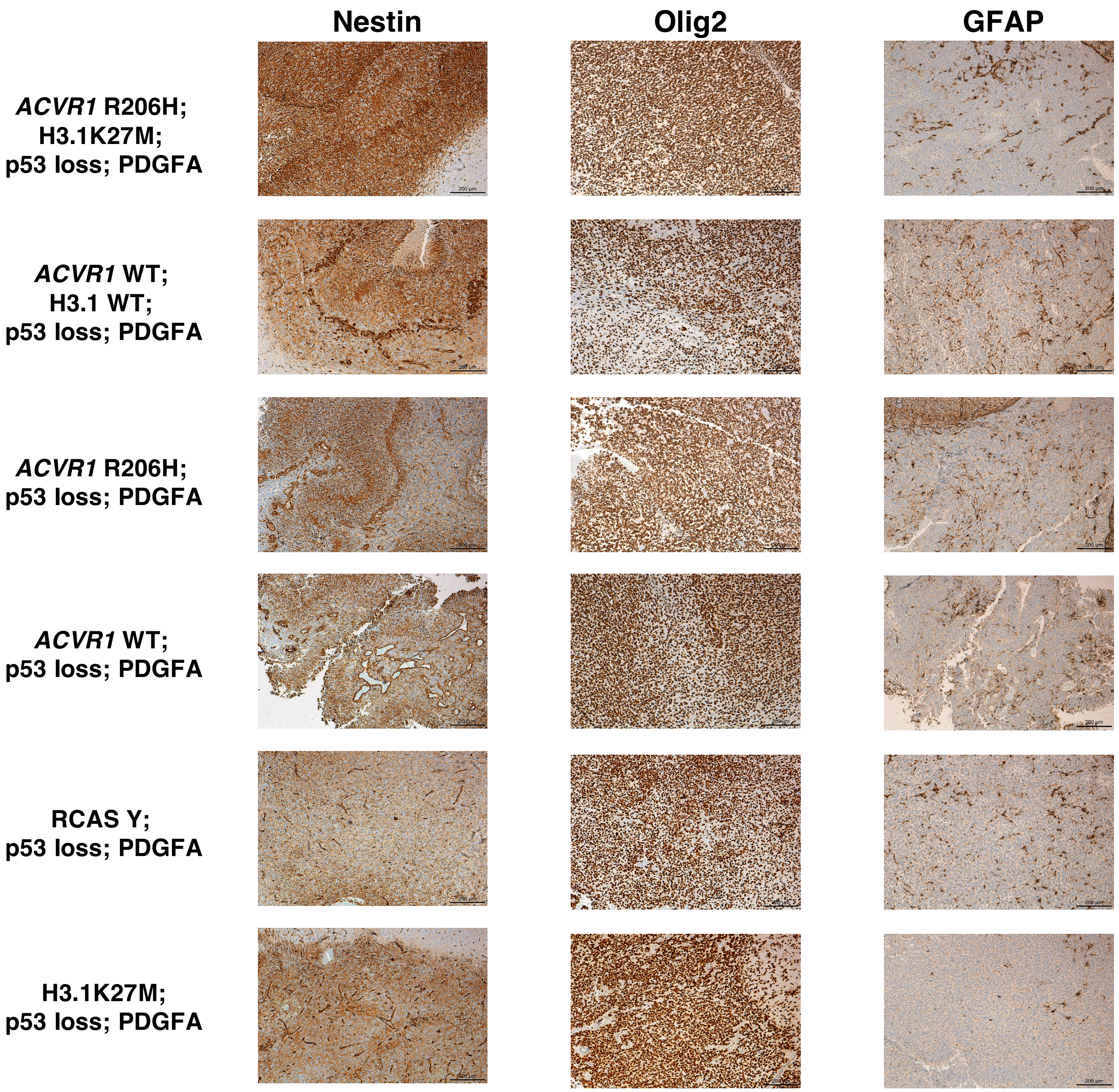

Supplementary Figure 3. Nestin, Olig2, and GFAP expression are not changed by *ACVR1* R206H. Representative IHC images of Nestin, Olig2, and GFAP from various PDGFA tumors. 10x magnification, scale bar = 200 μM.

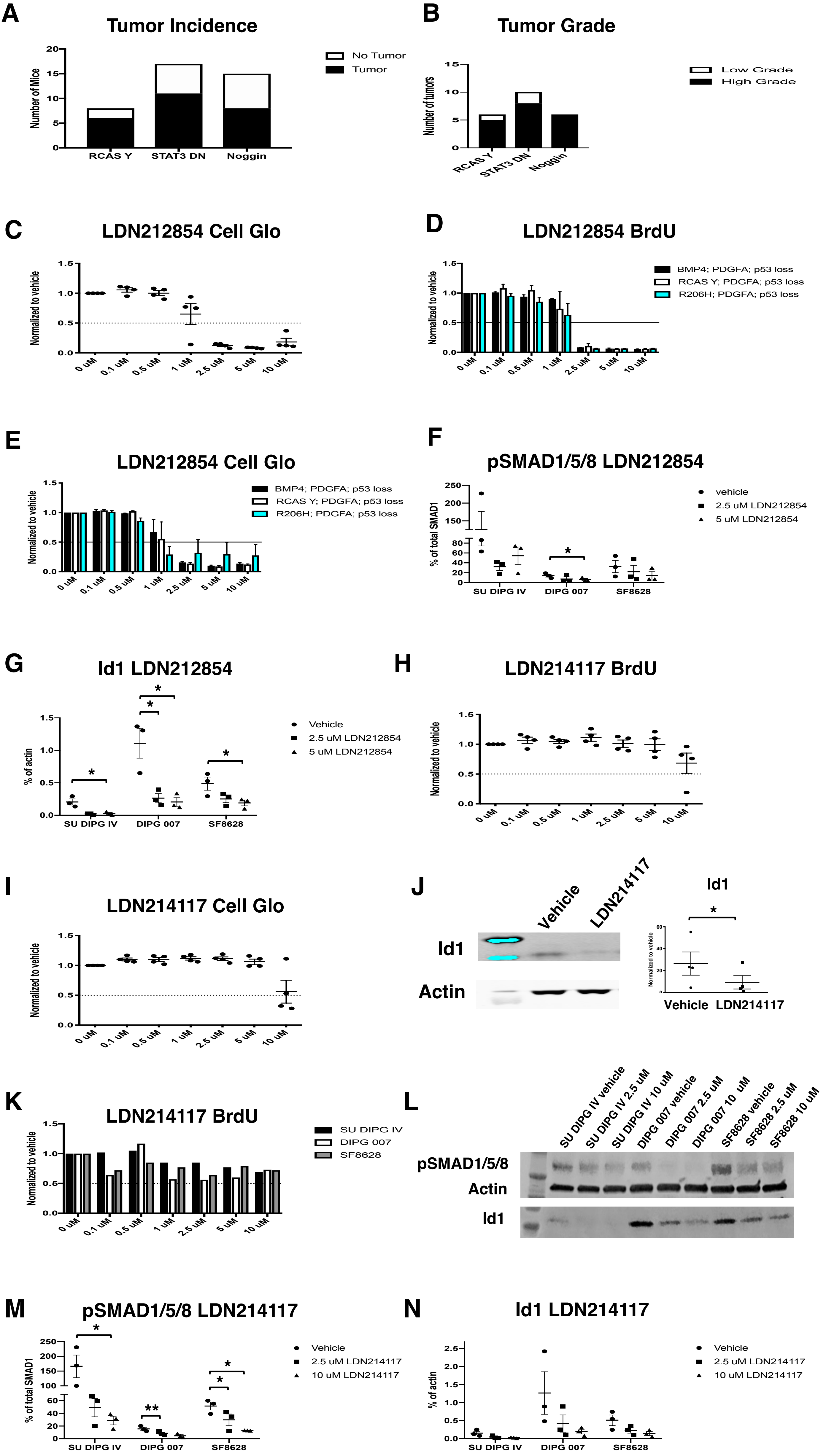
A
Tumor Incidence
Number of Mice
No Tumor
Tumor
RCAS Y
STAT3 DN
Noggin
B
Tumor Grade
Number of tumors
Low Grade
High Grade
RCAS Y
STAT3 DN
Noggin
C
LDN212854 Cell Glo
Normalized to vehicle
0 uM
0.1 uM
0.5 uM
1 uM
2.5 uM
5 uM
10 uM
D
LDN212854 BrdU
Normalized to vehicle
BMP4; PDGFA; p53 loss
RCAS Y; PDGFA; p53 loss
R206H; PDGFA; p53 loss
E
LDN212854 Cell Glo
Normalized to vehicle
BMP4; PDGFA; p53 loss
RCAS Y; PDGFA; p53 loss
R206H; PDGFA; p53 loss
F
pSMAD1/5/8 LDN212854
% of total SMAD1
vehicle
2.5 uM LDN212854
5 uM LDN212854
SU DIPG IV
DIPG 007
SF8628
G
Id1 LDN212854
% of actin
Vehicle
2.5 uM LDN212854
5 uM LDN212854
SU DIPG IV
DIPG 007
SF8628
H
LDN214117 BrdU
Normalized to vehicle
I
LDN214117 Cell Glo
Normalized to vehicle
J
Vehicle
LDN214117
Id1
Actin
Id1
Normalized to vehicle
Vehicle
LDN214117
K
LDN214117 BrdU
Normalized to vehicle
SU DIPG IV
DIPG 007
SF8628
L
SU DIPG IV vehicle
SU DIPG IV 2.5 uM
SU DIPG IV 10 uM
DIPG 007 vehicle
DIPG 007 2.5 uM
DIPG 007 10 uM
SF8628 vehicle
SF8628 2.5 uM
SF8628 10 uM
pSMAD1/5/8
Actin
Id1
M
pSMAD1/5/8 LDN214117
% of total SMAD1
Vehicle
2.5 uM LDN214117
10 uM LDN214117
SU DIPG IV
DIPG 007
SF8628
N
Id1 LDN214117
% of actin
Vehicle
2.5 uM LDN214117
10 uM LDN214117
SU DIPG IV
DIPG 007
SF8628

Supplementary Figure 4. *ACVR1* inhibitor LDN214117 does not inhibit proliferation or cell viability *in vitro*. (**a**) Tumor incidence of mice that were injected with RCAS-PDGFA, RCAS-Cre, RCAS-*ACVR1* R206H, and RCAS-Y (6/8 tumors = 75%) or RCAS-STAT3 DN (11/17 tumors = 65%) or RCAS-Noggin (8/15 tumors = 53%), Fisher's exact test. (**b**) Tumor grade from mice injected in (**a**), Fisher's exact test. (**c**) Tumor derived neurospheres from Nestin tv-a; $p53^{fl/fl}$ mice injected with RCAS-*ACVR1* R206H, RCAS-H3.1K27M, RCAS-Cre, and RCAS-PDGFA were treated *ACVR1* inhibitor LDN212854 for 24 hours and assessed for cell viability (n = 4). (**d** and **e**) All Ntv-a;$p53^{fl/fl}$ mice were injected with RCAS-PDGFA and RCAS-Cre along with additional RCAS viruses as indicated. Proliferation (**d**) and cell viability (**e**) of neurospheres incubated with LDN212854 for 24 hours (BMP4 lines n = 2, RCAS Y lines n = 2, and *ACVR1* R206H lines n = 4). (**f** and **g**) Western blot quantification of pSMAD1/5/8 **(f)** and Id1 **(g)** of human lines treated with LDN212854 from Fig. 6h (n = 3 independent experiments), * $p < 0.05$, paired t test. (**h**-**j**) Neurospheres were derived from primary tumors of RCAS-*ACVR1* R206H, RCAS-H3.1K27M, RCAS-Cre, and RCAS-PDGFA injected Nestin tv-a;$p53^{fl/fl}$ mice. Proliferation (**h**) and cell viability (**i**) of neurospheres treated with LDN214117 for 24 hours (n = 4). (**j**) Western blot analysis of Id1 expression of LDN214117 treated neurospheres versus vehicle neurospheres (n = 4), * $p < 0.05$, paired t test. (**k**-**n**) *ACVR1* mutant and wildtype human DIPG lines were treated with *ACVR1* inhibitor LDN214117 for 24 hours. (**k**) Proliferation of LDN214117 treated human lines (n = 3 independent experiments, triplicate wells). (**l**-**n**) Western blot analysis and quantification of pSMAD1/5/8 **(l and m)** and Id1 **(l and n)** expression of LDN214117 treated human lines (n = 3 independent experiments). All data represented as mean with SEM. * $p < 0.05$, paired t test.

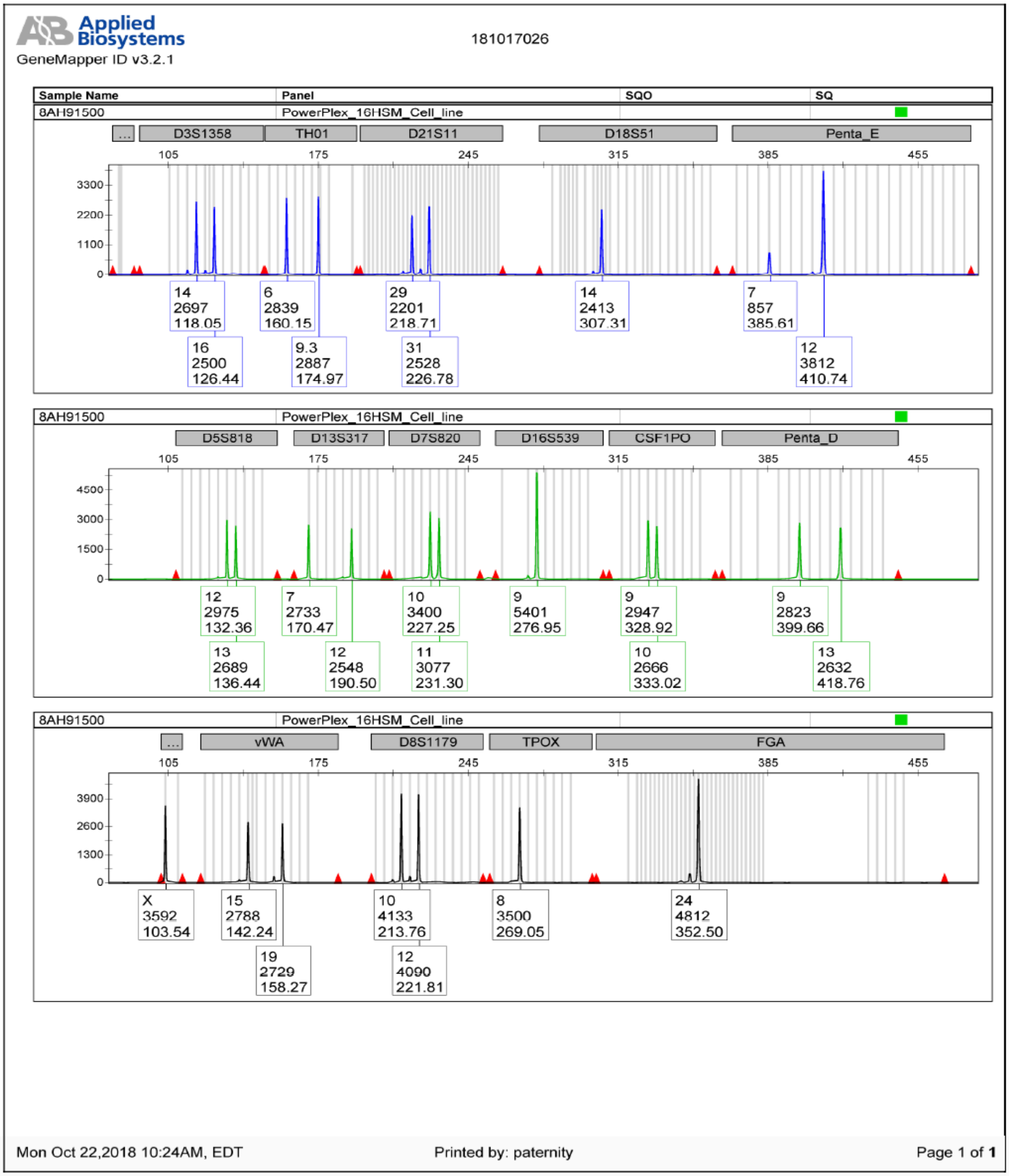

Supplementary Figure 5. Authentication of human DIPG cell line SU DIPG IV.

Electropherogram of STRs from human DIPG cell line SU DIPG IV. STR profiling results were obtained through PowerPlex 16HS + Mouse marker testing. Also see Supplementary Data 4.

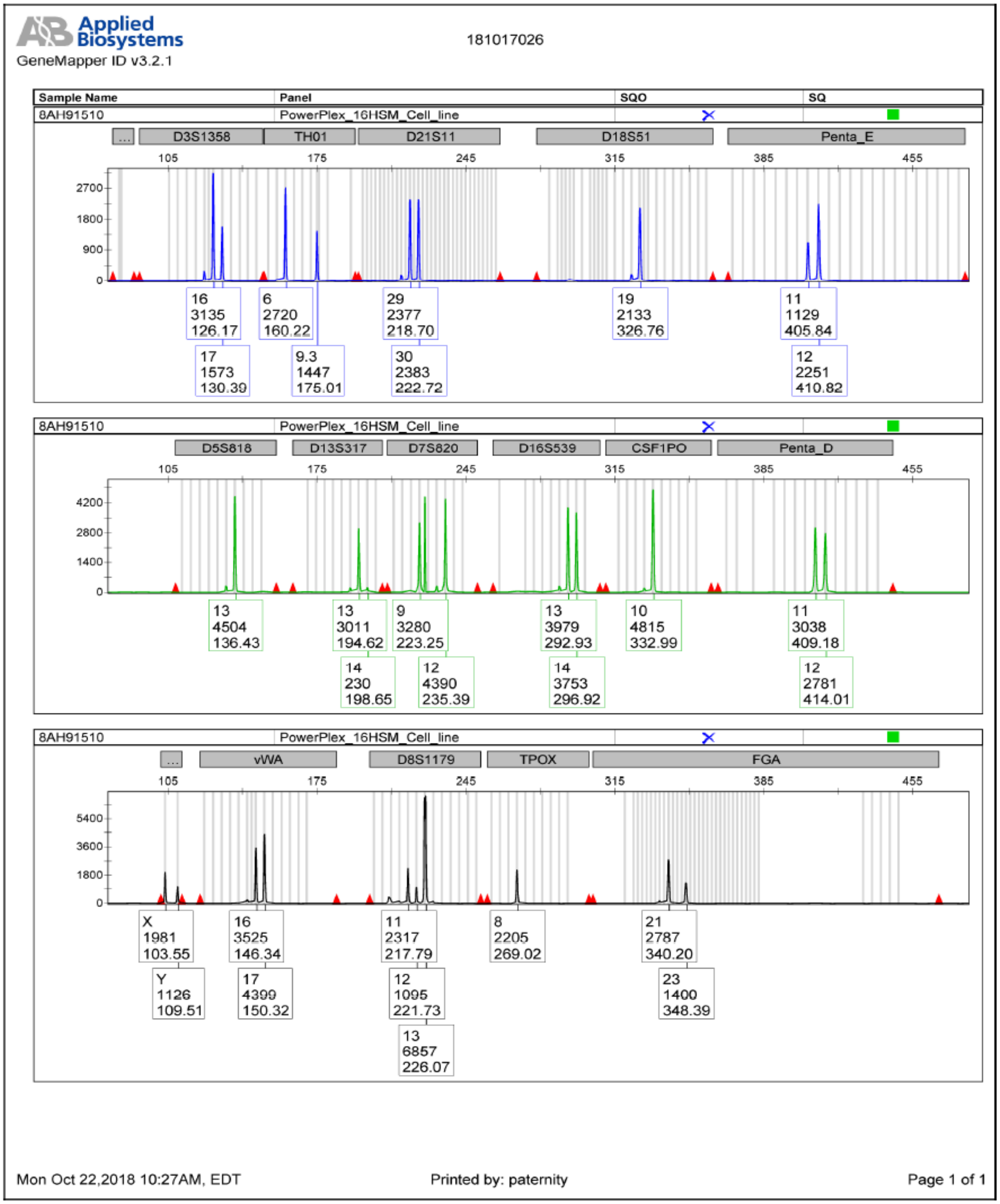

Supplementary Figure 6. Authentication of human DIPG cell line DIPG 007. Electropherogram of STRs from human DIPG cell line DIPG 007. STR profiling results were obtained through PowerPlex 16HS + Mouse marker testing. Also see Supplementary Data 4.

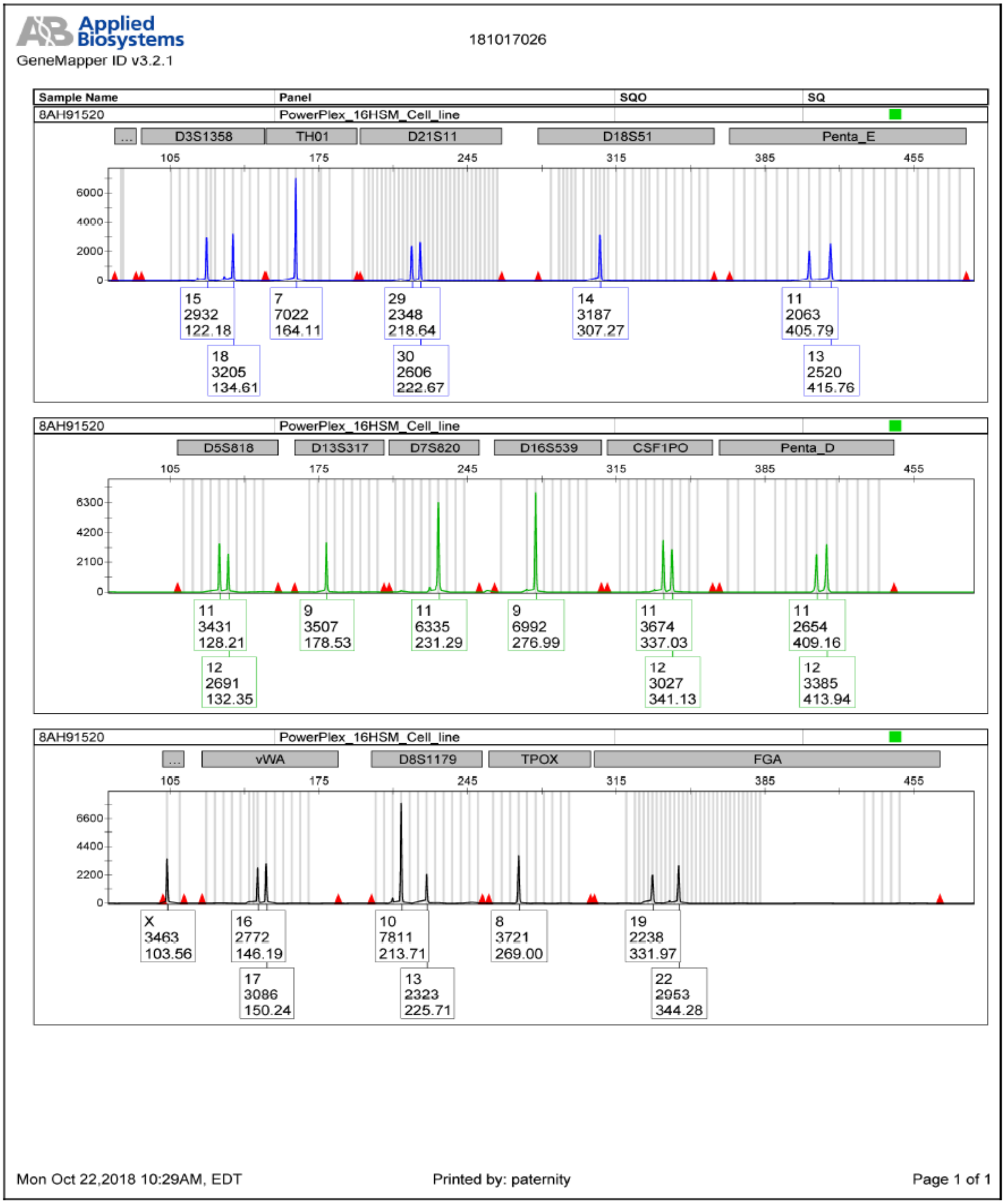

Supplementary Figure 7. Authentication of human DIPG cell line SF8628. Electropherogram of STRs from human DIPG cell line SF8628. STR profiling results were obtained through PowerPlex 16HS + Mouse marker testing. Also see Supplementary Data 4.

Supplementary Table 1 *ACVR1* virus combinations and lesion incidence *in vivo*

Virus Combination	Lesion Incidence
ACVR1 R206H, H3.1K27M, Cre (Ntv-a;p53$^{fl/fl}$)	4/10
ACVR1 R206H, H3.3K27M, Cre (Ntv-a;p53$^{fl/fl}$)	0/10
ACVR1 R206H, H3.1K27M, Cre (Ntv-a;p53$^{fl/fl}$;PTEN$^{fl/fl}$)	6/10
ACVR1 R206H, H3.1K27M, Cre, BMP4 (Ntv-a;p53$^{fl/fl}$)	3/6
ACVR1 R206H, H3.1 K27M, Cre, BMP4 (Ntv-a; p53$^{fl/fl}$;PTEN$^{fl/fl}$)	0/13
ACVR1 R206H, H3.1K27M, Cre, Activin A (Ntv-a;p53$^{fl/fl}$)	0/5
ACVR1 R206H, H3.1K27M, Cre, STAT3 WT (Ntv-a; p53$^{fl/fl}$)	0/14
ACVR1 R206H, H3.1K27M, Cre, STAT3 WT (Ntv-a;p53$^{fl/fl}$;PTEN$^{fl/fl}$)	0/4
ACVR1 R206H, H3.1K27M, Cre (B6 Ntv-a;PTEN^{fl})	0/12
ACVR1 G328V, H3.1K27M, Cre (Ntv-a;p53$^{fl/fl}$)	4/13
ACVR1 G328V, H3.1K27M, Cre (Ntv-a;p53$^{fl/fl}$;PTEN$^{fl/fl}$)	6/15
ACVR1 G328V, H3.1K27M, Cre, BMP4 (Ntv-a;p53$^{fl/fl}$)	0/7
ACVR1 G328E, H3.1K27M, Cre (Ntv-a;p53$^{fl/fl}$)	1/8
ACVR1 G328E, H3.1K27M, Cre (Ntv-a;p53$^{fl/fl}$;PTEN$^{fl/fl}$)	0/5
ACVR1 R206H only (Ntv-a;p53$^{fl/fl}$)	0/9
ACVR1 G328V only (Ntv-a;p53$^{fl/fl}$)	0/7
ACVR1 G328E only (Ntv-a;p53$^{fl/fl}$)	0/5
ACVR1 WT only (Ntv-a;p53$^{fl/fl}$)	0/3
H3.1K27M only (Ntv-a;p53$^{fl/fl}$)	0/4
ACVR1 R206H, H3.1K27M (Ntv-a;p53$^{fl/fl}$)	0/4
ACVR1 R206H, Cre (Ntv-a;p53$^{fl/fl}$)	0/6
H3.1K27M, Cre (Ntv-a;p53$^{fl/fl}$)	1/7
H3.1K27M, Cre (Ntv-a;p53$^{fl/fl}$;PTEN$^{fl/fl}$)	0/6
ACVR1 R206H, H3.1 WT, Cre Ntv-a;p53$^{fl/fl}$)	0/6
ACVR1 WT, H3.1K27M, Cre (Ntv-a;p53$^{fl/fl}$)	0/7

Supplementary Methods

Western blot antibodies

Antibody	Company
pSMAD1/5/8	Cell Signaling Technologies (#9511)
pSMAD1/5/8	Millipore (AB3848-I)
Total SMAD1	Cell Signaling Technologies (#9743)
Actin	Cell Signaling Technologies (#3700)
Actin (I-19)	Santa Cruz (sc-1616)
Id1	BioCheck (BCH-1/37-2)
Hes1	Cell Signaling Technologies (#11988)
CD31	Abcam (ab28364)
Phospho-Stat3 (Tyr705) (D3A7) XP	Cell Signaling Technologies (#9145)
Stat3 (D3Z2G)	Cell Signaling Technologies (#12640)

Antibodies for IF and IHC

Antibody	Company
Anti-phospho-histone H3	Millipore (#04-1093)
HA-probe (Y-11)	Santa Cruz (sc-805)
Nestin	BD Pharmigen (#556309)
Olig2	Millipore (AB9610)
GFAP	Dako (Z 0334)
CD44	BD Biosciences (#550538)
CD31	Abcam (ab28364)
pSTAT3 (Tyr705) (D3A7)	Cell Signaling Technologies (#9145)
Iba1	Wako (#019-19741)
Vimentin	Santa Cruz (sc-7557-R)
Anti-DDK	Origene (TA50011-100)

Primers for qRT-PCR

Gene	Forward (5'→3')	Reserve (5'→3')
**Actin**	TATTGGCAACGAGCGGTTCC	GGCATAGAGGTCTTTACGGATGTC
**Id1**	CCTAGCTGTTCGCTGAAGGC	CTCCGACAGACCAAGTACCAC
**Id2**	ATGAAAGCCTTCAGTCCGGTG	AGCAGACTCATCGGGTCGT
**Id3**	CTGTCGGAACGTAGCCTGG	GTGGTTCATGTCGTCCAAGAG
**Id4**	CCGCCCAACAAGAAAGTCAG	CCAGGATGTAGTCGATAACGTG
**L1Cam**	AAAGGTGCAAGGGTGACATTC	TCCCCACGTTCCTGTAGGT
**CD44**	TCGATTTGAATGTAACCTGCCG	CAGTCCGGGAGATACTGTAGC
**Snai2**	TGGTCAAGAAACATTTCAACGCC	GGTGAGGATCTCTGGTTTTGGTA
**Vimentin**	CGTCCACACGCACCTACAG	GGGGGATGAGGAATAGAGGCT
**TNC**	ACGGCTACCACAGAAGCTG	ATGGCTGTTGTTGCTATGGCA
**Sox10**	AGGTTGCTGAACGAAAGTGAC	CCGAGGTTGGTACTTGTAGTCC
