# Supplementary material for: ACVR1 R206H cooperates with H3.1K27M in promoting diffuse intrinsic pontine glioma pathogenesis
Source: Nat Commun. 2019 Mar 4;10:1023. doi: 10.1038/s41467-019-08823-9 (PMC6399349; doi:10.1038/s41467-019-08823-9)
Supplement: Supplementary file 3 — Description of Additional Supplementary Files [file 41467_2019_8823_MOESM3_ESM.pdf]

Description of Additional Supplementary Files

File Name: Supplementary Data 1
Description: Lists of significantly differentially expressed genes in ACVR1 R206H infected neurospheres compared to ACVR1 WT infected neurospheres, with and without H3.1K27M, as determined by RNAseq analysis.

File Name: Supplementary Data 2
Description: GSEA analysis results of ACVR1 R206H infected neurospheres compared to ACVR1 WT infected neurospheres, with and without H3.1K27M, as determined by RNAseq analysis.

File Name: Supplementary Data 3
Description: Uncropped and unprocessed Western blot gels of representative images.

File Name: Supplementary Data 4
Description: Validation of human cells lines using short tandem repeat analysis
