# Supplementary material for: ACVR1 R206H cooperates with H3.1K27M in promoting diffuse intrinsic pontine glioma pathogenesis
Source: Nat Commun. 2019 Mar 4;10:1023. doi: 10.1038/s41467-019-08823-9 (PMC6399349; doi:10.1038/s41467-019-08823-9)

Hoeman et al. Figure 1 Western blots

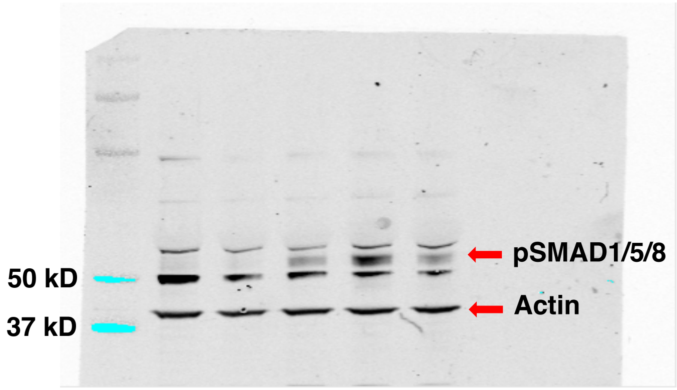

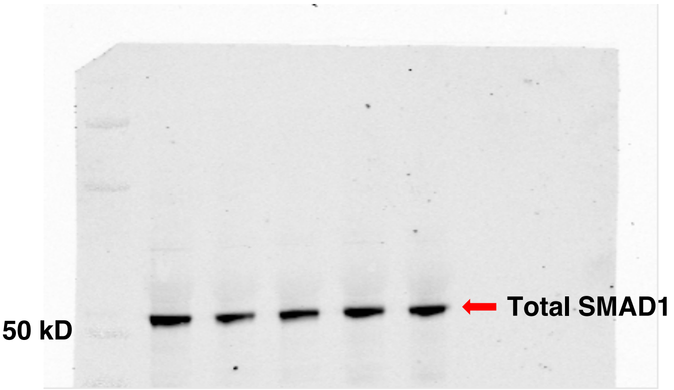

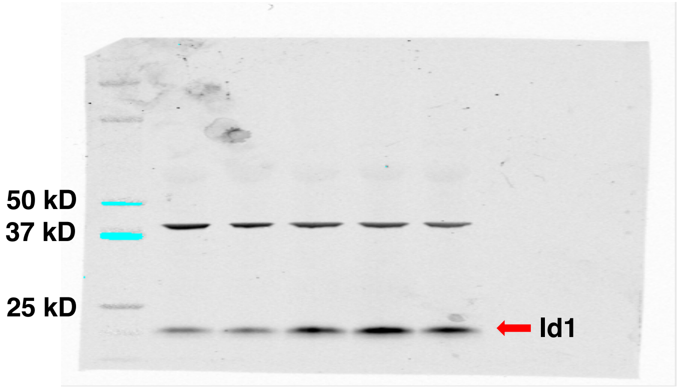

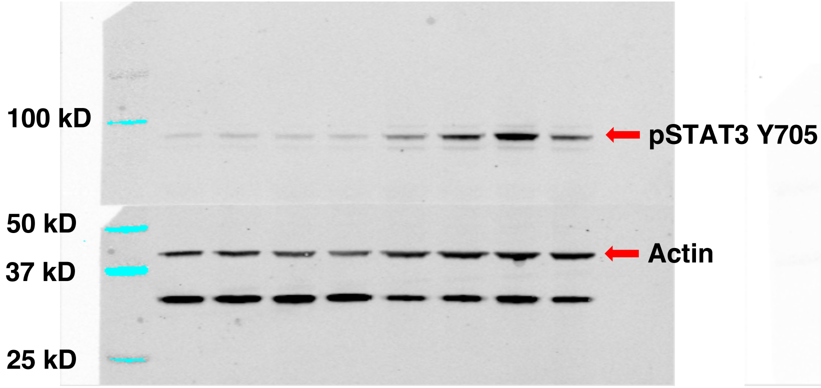

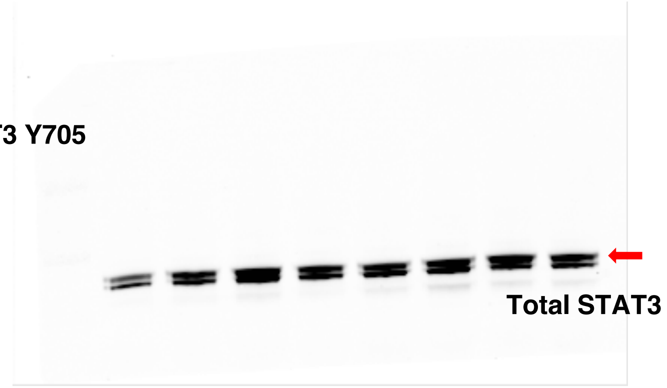

Hoeman et al. Figure 5 Western blots

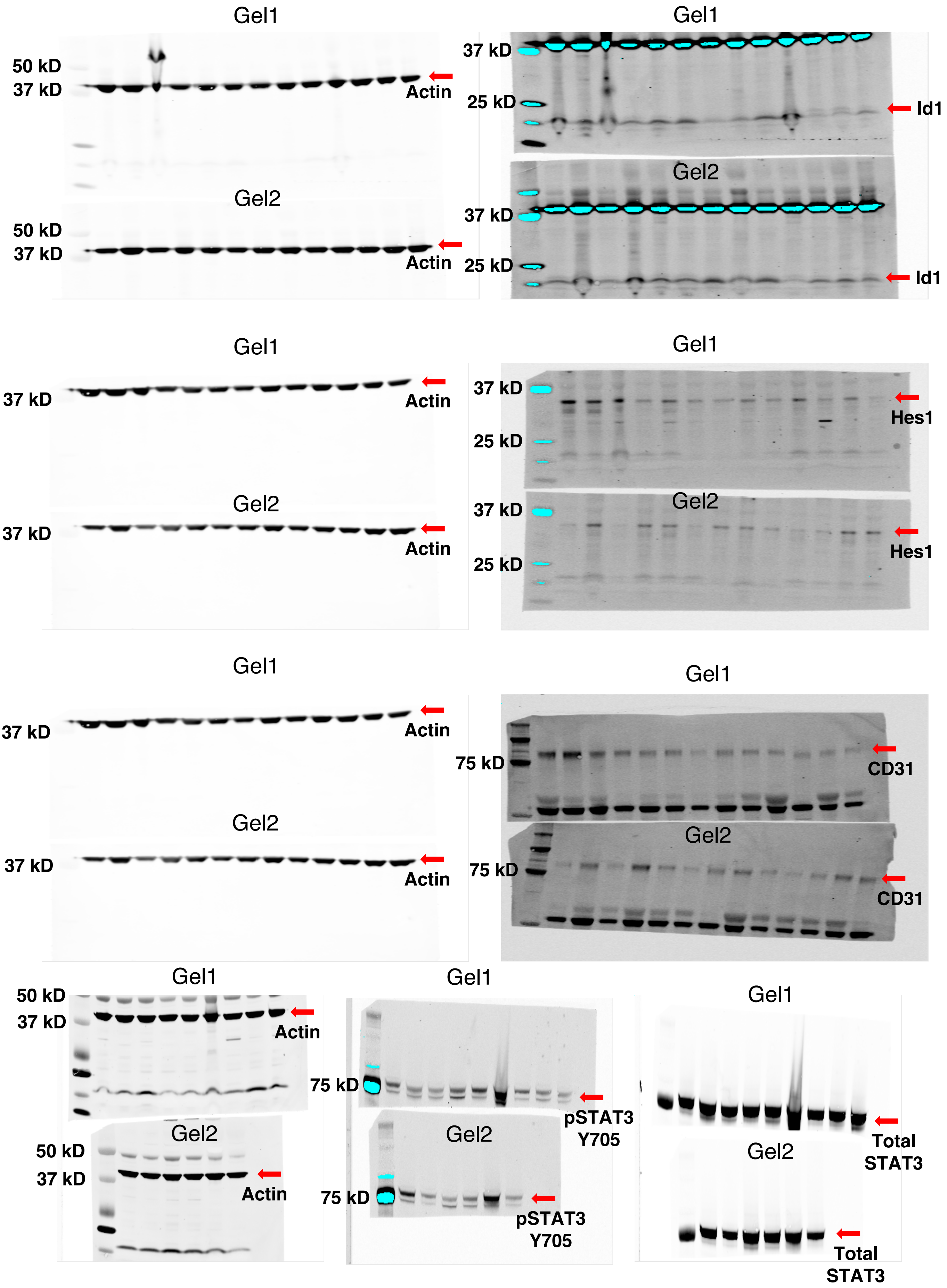

Hoeman et al. Figure 6 Western blots

Human Lysates

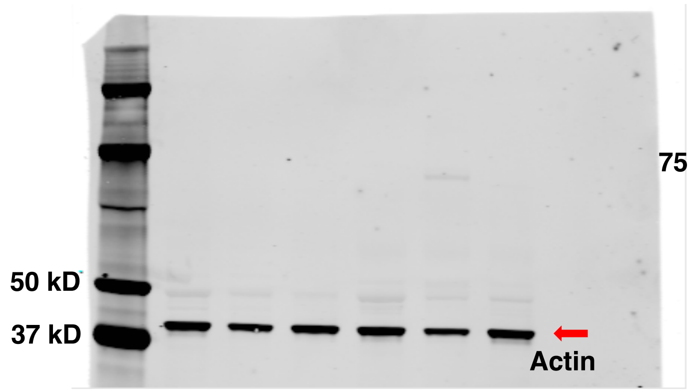

Human Lysates

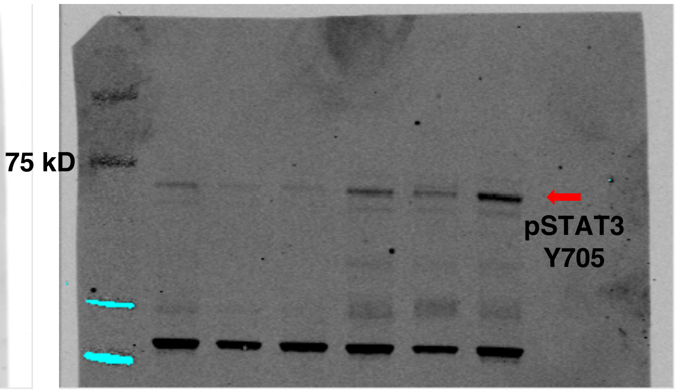

Human Lysates

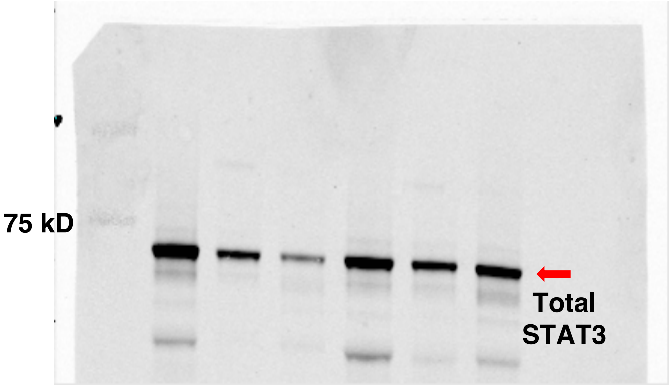

LDN212854 Treated Lines (Mouse)

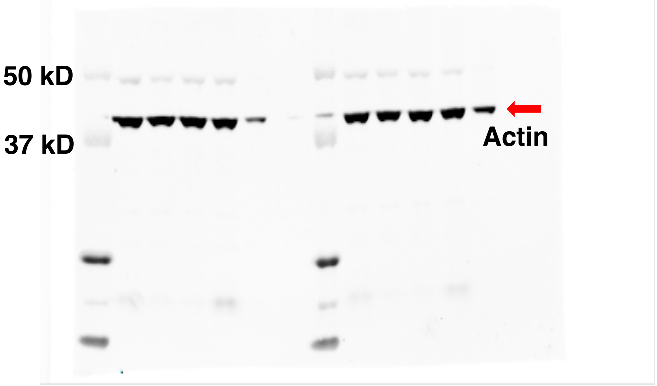

LDN212854 Treated Lines (Mouse)

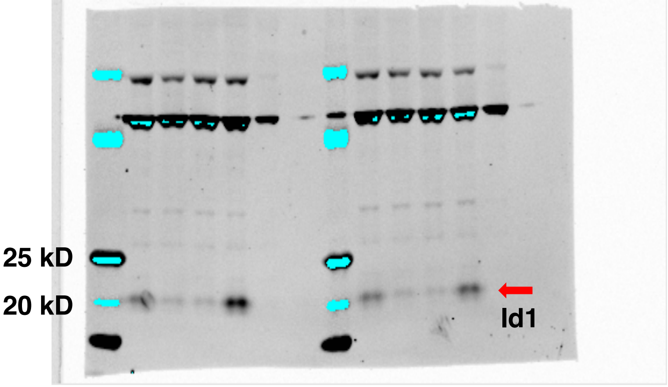

LDN212854 Treated Lines (Human)

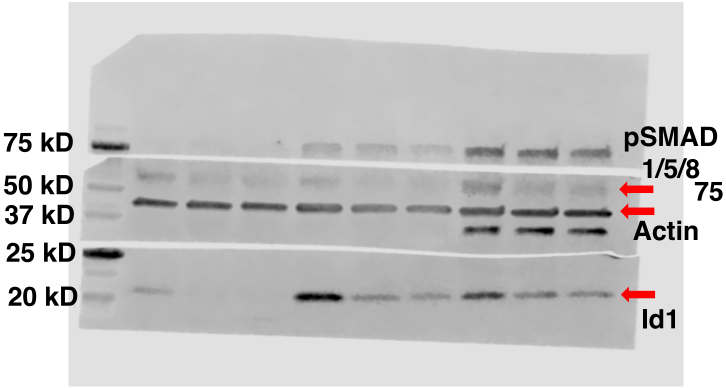

LDN212854 Treated Lines (Human)

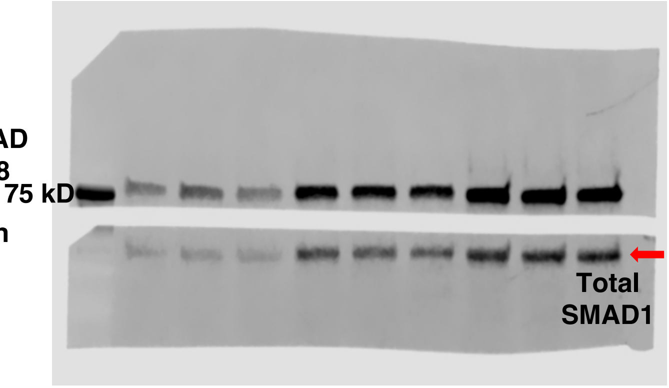

Hoeman et al. Supplementary Figure 1 Western blots

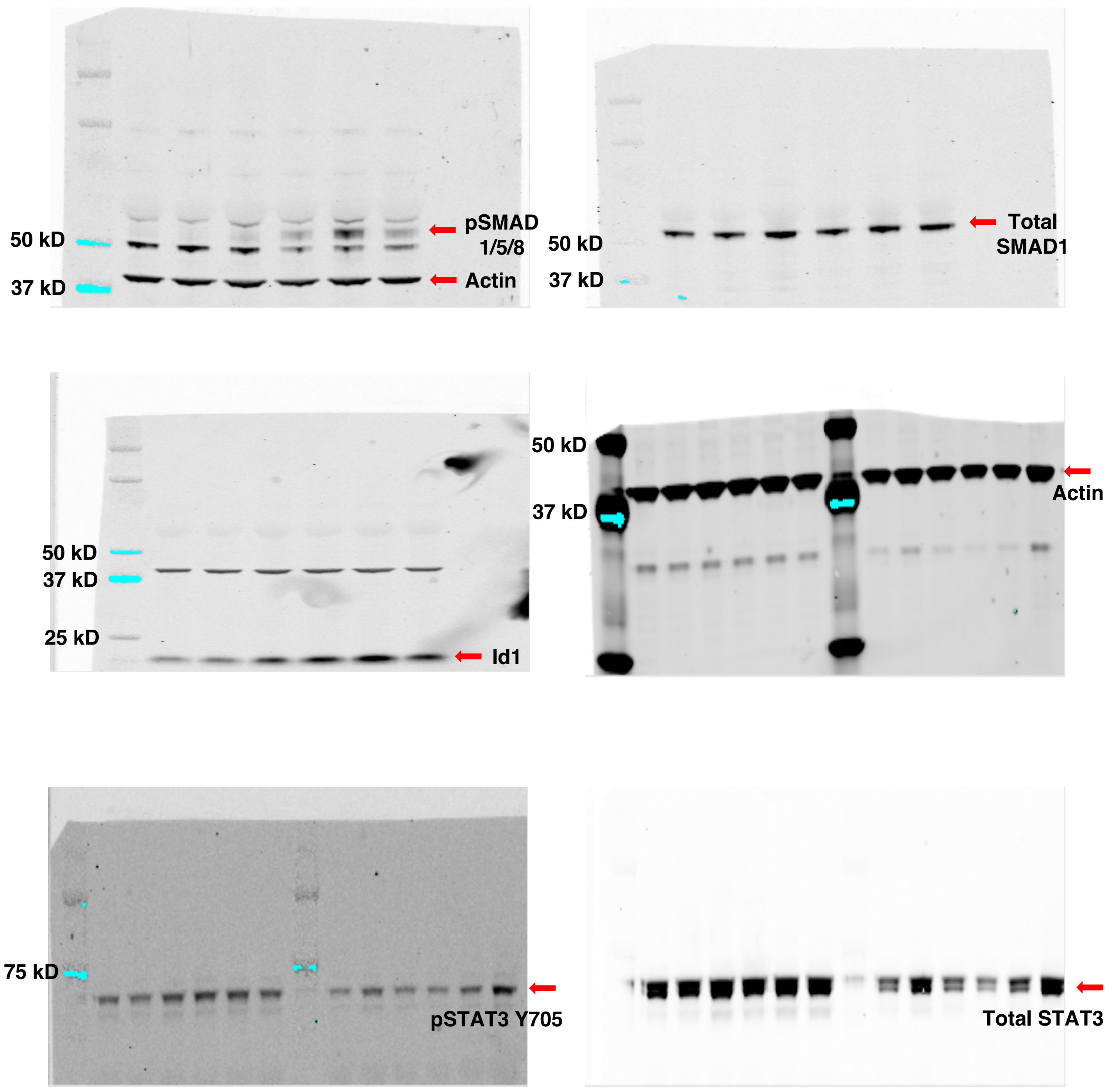

Hoeman et al. Supplementary Figure 4 Western blots

LDN214117 Treated Lines (Mouse)

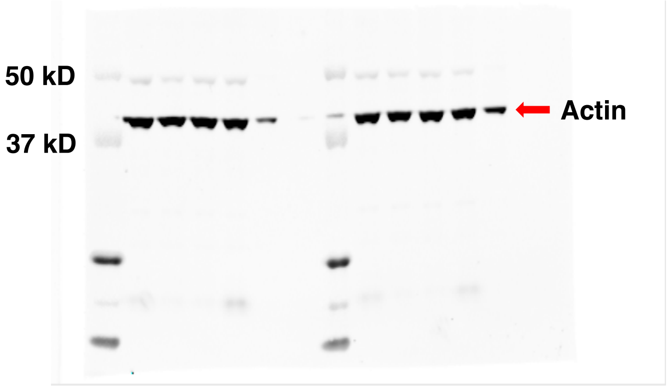

LDN214117 Treated Lines (Mouse)

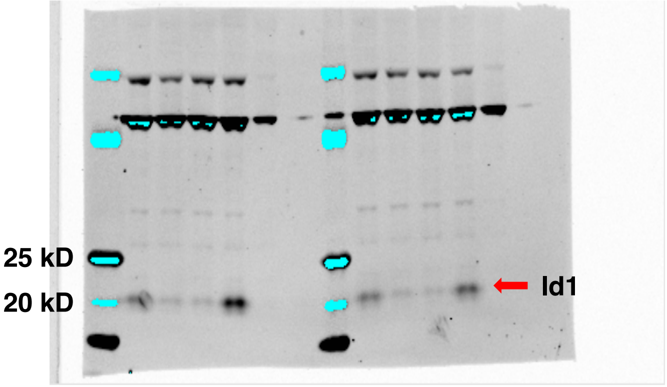

LDN214117 Treated Lines (Human)

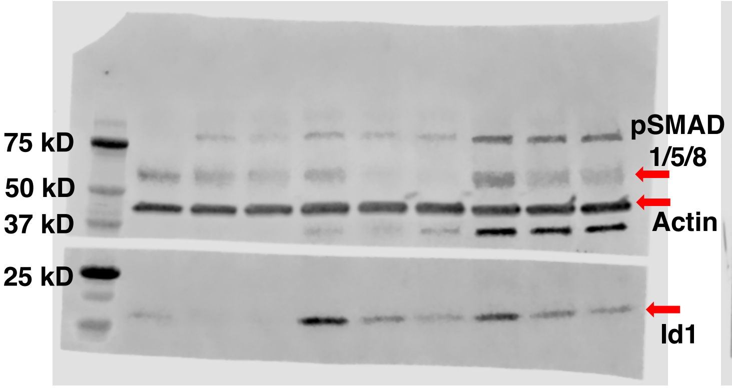

LDN214117 Treated Lines (Human)

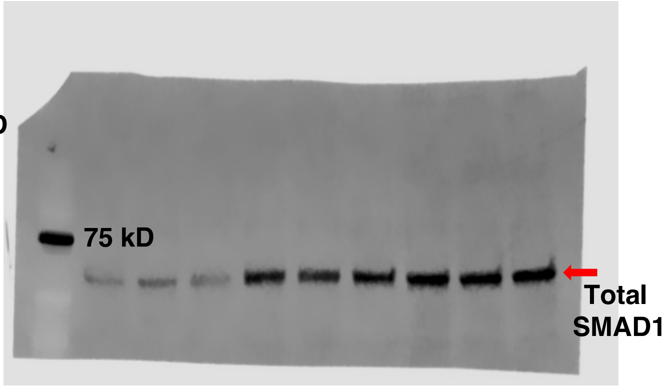

Supplement: Supplementary file 6 — Supplementary Data 3 [file 41467_2019_8823_MOESM6_ESM.pdf]
