# Supplementary material for: ACVR1 R206H cooperates with H3.1K27M in promoting diffuse intrinsic pontine glioma pathogenesis
Source: Nat Commun. 2019 Mar 4;10:1023. doi: 10.1038/s41467-019-08823-9 (PMC6399349; doi:10.1038/s41467-019-08823-9)
Supplement: Supplementary file 8 — Reporting Summary [file 41467_2019_8823_MOESM8_ESM.pdf]

nature research

Corresponding author(s): Oren Becher

Last updated by author(s): Jan 28, 2019

Reporting Summary

Nature Research wishes to improve the reproducibility of the work that we publish. This form provides structure for consistency and transparency in reporting. For further information on Nature Research policies, see Authors & Referees and the Editorial Policy Checklist.

Statistics

For all statistical analyses, confirm that the following items are present in the figure legend, table legend, main text, or Methods section.

n/a	Confirmed	
☐	☒	The exact sample size (*n*) for each experimental group/condition, given as a discrete number and unit of measurement
☐	☒	A statement on whether measurements were taken from distinct samples or whether the same sample was measured repeatedly
☐	☒	The statistical test(s) used AND whether they are one- or two-sided<br>*Only common tests should be described solely by name; describe more complex techniques in the Methods section.*
☐	☒	A description of all covariates tested
☐	☒	A description of any assumptions or corrections, such as tests of normality and adjustment for multiple comparisons
☐	☒	A full description of the statistical parameters including central tendency (e.g. means) or other basic estimates (e.g. regression coefficient) AND variation (e.g. standard deviation) or associated estimates of uncertainty (e.g. confidence intervals)
☐	☒	For null hypothesis testing, the test statistic (e.g. *F*, *t*, *r*) with confidence intervals, effect sizes, degrees of freedom and *P* value noted<br>*Give P values as exact values whenever suitable.*
☒	☐	For Bayesian analysis, information on the choice of priors and Markov chain Monte Carlo settings
☒	☐	For hierarchical and complex designs, identification of the appropriate level for tests and full reporting of outcomes
☒	☐	Estimates of effect sizes (e.g. Cohen's *d*, Pearson's *r*), indicating how they were calculated

Our web collection on statistics for biologists contains articles on many of the points above.

Software and code

Policy information about availability of computer code

Data collection	No software was used.
Data analysis	Data analysis was performed using GraphPad Prism software (Version 7.0).

For manuscripts utilizing custom algorithms or software that are central to the research but not yet described in published literature, software must be made available to editors/reviewers. We strongly encourage code deposition in a community repository (e.g. GitHub). See the Nature Research guidelines for submitting code & software for further information.

Data

Policy information about availability of data

All manuscripts must include a data availability statement. This statement should provide the following information, where applicable:

- Accession codes, unique identifiers, or web links for publicly available datasets
- A list of figures that have associated raw data
- A description of any restrictions on data availability

RNAseq data that support the findings of this study have been deposited in Geo Bank with Geo accession number GSE125627. The authors declare that data supporting the findings of this study are available within this published article and its supplementary information files. Data that supports the findings of this paper are available from the corresponding author upon reasonable request.

Field-specific reporting

Please select the one below that is the best fit for your research. If you are not sure, read the appropriate sections before making your selection.

☒ Life sciences ☐ Behavioural & social sciences ☐ Ecological, evolutionary & environmental sciences

For a reference copy of the document with all sections, see nature.com/documents/nr-reporting-summary-flat.pdf

Life sciences study design

All studies must disclose on these points even when the disclosure is negative.

Sample size	For in vitro experiments, no sample size calculation was performed. In this case, a minimum of 3 biologically different samples were used for statistical analysis. It should be noted that in most cases, a minimum of 5 biologically different samples were used. Sample size for in vivo studies were based on power analysis that was performed previously in our lab using our this mouse modeling approach (RCAS/tv-a)
Data exclusions	All data was included for analysis, except for determining tumor grade. In this case, only tumors from euthanized animals were included for analysis. Tumors from found dead animals were excluded as to avoid false positives.
Replication	To verify the reproducibility of the experimental findings, in vitro experiments were plated in triplicate wells and performed for a minimum of 3 separate experiments. For in vivo studies, a minimum of 3 infected litters with viral combination were performed. All attempts at replication were successful.
Randomization	Randomization was performed primarily for in vivo drug studies. Mice that were symptomatic at approximately the same age were included for these studies, and when possible, would be from the same litter.
Blinding	Tumor presence and grading were determined by a blinded neuropathologist who was not aware which viral combination was used to infect the mouse brain to induce brain tumors

Reporting for specific materials, systems and methods

We require information from authors about some types of materials, experimental systems and methods used in many studies. Here, indicate whether each material, system or method listed is relevant to your study. If you are not sure if a list item applies to your research, read the appropriate section before selecting a response.

Materials & experimental systems

n/a	Involved in the study	
☐	☒	Antibodies
☐	☒	Eukaryotic cell lines
☒	☐	Palaeontology
☐	☒	Animals and other organisms
☐	☒	Human research participants
☒	☐	Clinical data

Methods

n/a	Involved in the study	
☒	☐	ChIP-seq
☒	☐	Flow cytometry
☐	☒	MRI-based neuroimaging

Antibodies

Antibodies used	pSMAD1/5/8 Cell Signaling Technologies (#9511), pSMAD1/5/8 Millipore (AB3848-I), Total SMAD1 Cell Signaling Technologies (#9743), Actin Cell Signaling Technologies (#3700), Actin (I-19) Santa Cruz (sc-1616), Id1 BioCheck (BCH-1/37-2), Hes1 Cell Signaling Technologies (#11988), CD31 Abcam (ab28364), Phospho-Stat3 (Tyr705) (D3A7) XP Cell Signaling Technologies (#9145), Stat3 (D3Z2G) Cell Signaling Technologies (#12640)
Validation	All antibodies used in this paper were validated by the manufacturer and have been cited in previous publications.

Eukaryotic cell lines

Policy information about cell lines

Cell line source(s)	Cell lines were obtained from Rintaro Hashizume
Authentication	STR authentification was performed
Mycoplasma contamination	we confirm that all cell lines were tested for mycoplasma and were negative.

Commonly misidentified lines (See ICLAC register)	none

Animals and other organisms

Policy information about studies involving animals; ARRIVE guidelines recommended for reporting animal research

Laboratory animals	Nestin-Tv-a (Ntv-a);p53fl/fl and Ntv-a; p53fl/fl;PTENfl/fl mice were used in this paper. Both female and male mice were included in data analysis. The use of neonatal (postnatal day 3) and adult mice were used for experiments.
Wild animals	This study did not involve wild animals.
Field-collected samples	This study did not involve samples collected from the field.
Ethics oversight	All animals were used according to protocols approved at Duke University and Northwestern University Animal Care and Use Committee and the Guide for the Care and Use of Laboratory Animals (Animal Protocol A214-13-8 at Duke University and IS00005105 at Northwestern University).

Note that full information on the approval of the study protocol must also be provided in the manuscript.

Human research participants

Policy information about studies involving human research participants

Population characteristics	DIPG is a rare childhood brain tumor. Approximately 250 children get diagnosed with DIPG each year in the United States.
Recruitment	DIPG is a rare tumor. Families of children with DIPG who were being treated at Children's National Medical Center were asked to consider tumor donation via autopsy when the treatment failed as DIPG is incurable
Ethics oversight	Children's National Medical Center, IRB approved protocol, IRB-1339

Note that full information on the approval of the study protocol must also be provided in the manuscript.

Magnetic resonance imaging

Experimental design

Design type	MRI was used to help diagnose which mice had tumors.
Design specifications	Starting at 6 weeks post infection mice were scheduled for MRI to determine if a tumor was present
Behavioral performance measures	No behavioral performances were measured.

Acquisition

Imaging type(s)	structural
Field strength	7 Tesla
Sequence & imaging parameters	10 minutes prior to scanning each mouse was injected intraperitoneally with gadolinium based MR contrast agent at 0.3mmol/kg to allow agent to reach the brain and enhance the tumor regions. Each mouse was then anesthesized using a mixture of isoflurane and 100% O2 and placed in an MR compatible cradle. MR images were acquired using a dedicated four channel mouse brain coil (Bruker, Germany). After initial localization sequences (tri-axial gradient echo sequences) a series of 2D images were acquired using T2 weighted Multi Spin Echo sequences in all 3 directions (transverse, longitudinal, and sagittal) with TR=2000 msec and TE= 40 msec with a spatial resolution of approximately 80 microns and slice thickness of 0.7mm. Finally, a 3D gradient T1 weighted echo sequence with isotropic resolution 150 microns was acquired with TR=40 msec, TE= 3msec and Flip Angle FA = 10 yielding high resolution 3D picture of the brain of each mouse. Post-acquisition inspection of the set of 2D and 3D images enabled assessment of morphological abnormalities associated with detection of tumor masses
Area of acquisition	Brain

Diffusion MRI ☐ Used ☒ Not used

Preprocessing

Preprocessing software	Not applicable
Normalization	Data was not normalized.

Normalization template	*Describe the template used for normalization/transformation, specifying subject space or group standardized space (e.g. original Talairach, MNI305, ICBM152) OR indicate that the data were not normalized.*
Noise and artifact removal	Noise and artifact were not removed
Volume censoring	No volume censoring was performed

Statistical modeling & inference

Model type and settings	No statistical modeling was used.
Effect(s) tested	MRI was strictly used to determine if a tumor was present for the preclinical trial

Specify type of analysis: ☒ Whole brain ☐ ROI-based ☐ Both

Statistic type for inference (See Eklund et al. 2016)	*Specify voxel-wise or cluster-wise and report all relevant parameters for cluster-wise methods.*
Correction	*Describe the type of correction and how it is obtained for multiple comparisons (e.g. FWE, FDR, permutation or Monte Carlo).*

Models & analysis

n/a	Involved in the study
☒	☐ Functional and/or effective connectivity
☒	☐ Graph analysis
☒	☐ Multivariate modeling or predictive analysis
